# Genotyping of Human Lice Suggests Multiple Emergences of Body Lice from Local Head Louse Populations

**DOI:** 10.1371/journal.pntd.0000641

**Published:** 2010-03-23

**Authors:** Wenjun Li, Gabriel Ortiz, Pierre-Edouard Fournier, Gregory Gimenez, David L. Reed, Barry Pittendrigh, Didier Raoult

**Affiliations:** 1 URMITE, UMR CNRS 6236, IRD 198, Université de la Méditerranée, Faculté de Médecine, Marseille, France; 2 Florida Museum of Natural History, Dickinson Hall, University of Florida, Gainesville, Florida, United States of America; 3 Department of Entomology, University of Illinois at Urbana-Champaign, Urbana, Illinois, United States of America; Institut Pasteur, France

## Abstract

**Background:**

Genetic analyses of human lice have shown that the current taxonomic classification of head lice (*Pediculus humanus capitis*) and body lice (*Pediculus humanus humanus*) does not reflect their phylogenetic organization. Three phylotypes of head lice A, B and C exist but body lice have been observed only in phylotype A. Head and body lice have different behaviours and only the latter have been involved in outbreaks of infectious diseases including epidemic typhus, trench fever and louse borne recurrent fever. Recent studies suggest that body lice arose several times from head louse populations.

**Methods and Findings:**

By introducing a new genotyping technique, sequencing variable intergenic spacers which were selected from louse genomic sequence, we were able to evaluate the genotypic distribution of 207 human lice. Sequence variation of two intergenic spacers, S2 and S5, discriminated the 207 lice into 148 genotypes and sequence variation of another two intergenic spacers, PM1 and PM2, discriminated 174 lice into 77 genotypes. Concatenation of the four intergenic spacers discriminated a panel of 97 lice into 96 genotypes. These intergenic spacer sequence types were relatively specific geographically, and enabled us to identify two clusters in France, one cluster in Central Africa (where a large body louse outbreak has been observed) and one cluster in Russia. Interestingly, head and body lice were not genetically differentiated.

**Conclusions:**

We propose a hypothesis for the emergence of body lice, and suggest that humans with both low hygiene and head louse infestations provide an opportunity for head louse variants, able to ingest a larger blood meal (a required characteristic of body lice), to colonize clothing. If this hypothesis is ultimately supported, it would help to explain why poor human hygiene often coincides with outbreaks of body lice. Additionally, if head lice act as a reservoir for body lice, and that any social degradation in human populations may allow the formation of new populations of body lice, then head louse populations are potentially a greater threat to humans than previously assumed.

## Author Summary

While being phenotypically and physiologically different, human head and body lice are indistinguishable based on mitochondrial and nuclear genes. As protein-coding genes are too conserved to provide significant genetic diversity, we performed strain-typing of a large collection of human head and body lice using variable intergenic spacer sequences. Ninety-seven human lice were classified into ninety-six genotypes based on four intergenic spacer sequences. Genotypic and phylogenetic analyses using these sequences suggested that human head and body lice are still indistinguishable. We hypothesized that the phenotypic and physiological differences between human head and body lice are controlled by very limited mutations. Under conditions of poor hygiene, head lice can propagate very quickly. Some of them will colonize clothing, producing a body louse variant (genetic or phenetic), which can lead to an epidemic. Lice collected in Rwanda and Burundi, where outbreaks of louse-borne diseases have been recently reported, are grouped tightly into a cluster and those collected from homeless people in France were also grouped into a cluster with lice collected in French non-homeless people. Our strain-typing approach based on highly variable intergenic spacers may be helpful to elucidate louse evolution and to survey louse-borne diseases.

## Introduction

Lice are extremely well-adapted ectoparasites that are usually host-specific [1]. Three recognized taxa of lice feed on humans: head lice (*Pediculus humanus capitis*), body lice (*Pediculus humanus humanus*), and pubic lice (*Pthirius pubis*), feed on humans. As one of the most intimate parasites of humans, lice have been widely used as a genetic model to infer host evolutionary history by providing genetic date independent of host data [1],[2]. Several nuclear and mitochondrial DNA sequences have previously been used in population genetic studies of human lice. Of these, the nuclear DNA sequences, *EF-1α* and 18S rDNA, discriminated lice into two subgroups, lice from Sub-Saharan Africa and lice worldwide[3]. In each subgroup, the head lice were genetically different from the body lice [3]. However, Leo *et al.* suggested that 18S rDNA phylogeny was concordant with the phylogenies from mitochondrial genes, but the *EF-1α* phylogeny was concordant neither with the mitochondrial phylogenies nor with the 18S rRNA phylogeny [4]. Furthermore, the mitochondrial DNA markers, partial *COI* and *cytB* classified the lice into three deeply divergent clades (Clades A, B, and C), and each having unique geographical distribution. Clade A includes both head and body lice and is worldwide in distribution. Clade B consists only of head lice from America, Australia and Europe, and Clade C consists only of lice from Ethiopia and Nepal [5].

More variable genetic markers, such as internal transcribed spacers (*ITS*) of ribosomal DNA and microsatellite DNA, were also used to deduce the louse phylogeny. However, the *ITS* that was chosen was not useful to study the populations structure of human lice because some of the lice had more than one copy of *ITS2* in their genome [6]. A subsequent microsatellite DNA-based study has suggested that human head and body lice are genetically distinct [7], however recent studies contradict this hypothesis [5],[8]. Taken together, the population structure of human lice is complex and still unclear. The previously used genetic markers were mostly mitochondrial and nuclear genes that were too conserved to generate more information of genetic diversity of studied louse isolates. So far, no genetic marker has been found that can discriminate among individual human lice.

While being used as a suitable genetic model to study the evolutionary history of humans, lice have long been associated with infectious diseases. Of the three types of lice associated with humans, body lice can be a serious public health problem because they are known vectors of *Rickettsia prowazekii*, *Bartonella quintana*, and *Borrelia recurrentis*, which cause epidemic typhus, trench fever and relapsing fever in humans, respectively [9]. However, medical interest in louse-borne diseases had waned for more than 30 years until 1997, when an outbreak of infection of louse-transmissed *R. prowazekii* and *B. quintana* occurred among the displaced population of Burundi [10],[11].

Body lice have long been recognized as human parasites and although typically prevalent in rural communities in upland areas of countries close to the equator, high incidence of louse-borne infections are also increasingly found in the homeless in developed countries [9],[12],[13]. In contrast, head lice represent a major economic and social concern throughout developed nations, because head louse infestations are often associated with school-aged children.

Faster evolving molecular markers are needed in order to epidemiologically survey the vectors of these bacterial infections and to address more recent population-level questions, [8],[14]. Among these fast-evolving genetic markers, intergenic spacers are promising for individual discrimination of lice because they are under less evolutionary pressure, and are more variable than coding genes. These factors make intergenic spacers useful for understanding the population genetics of lice. Highly variable intergenic spacers are useful for strain-typing many bacteria, including louse-transmitted *R. prowazekii* and *B. quintana* [15],[16] as well as other pathogenic bacteria [17]. Additionally, intergenic spacer sequences for individual discrimination of lice, are now publicly available [14]. In this study, we used four highly variable intergenic spacers that were selected from the genomic sequence to study the genotypic distribution of a large collection of lice of worldwide origins.

## Methods

### Louse collection and DNA preparation

**Figure 1:**
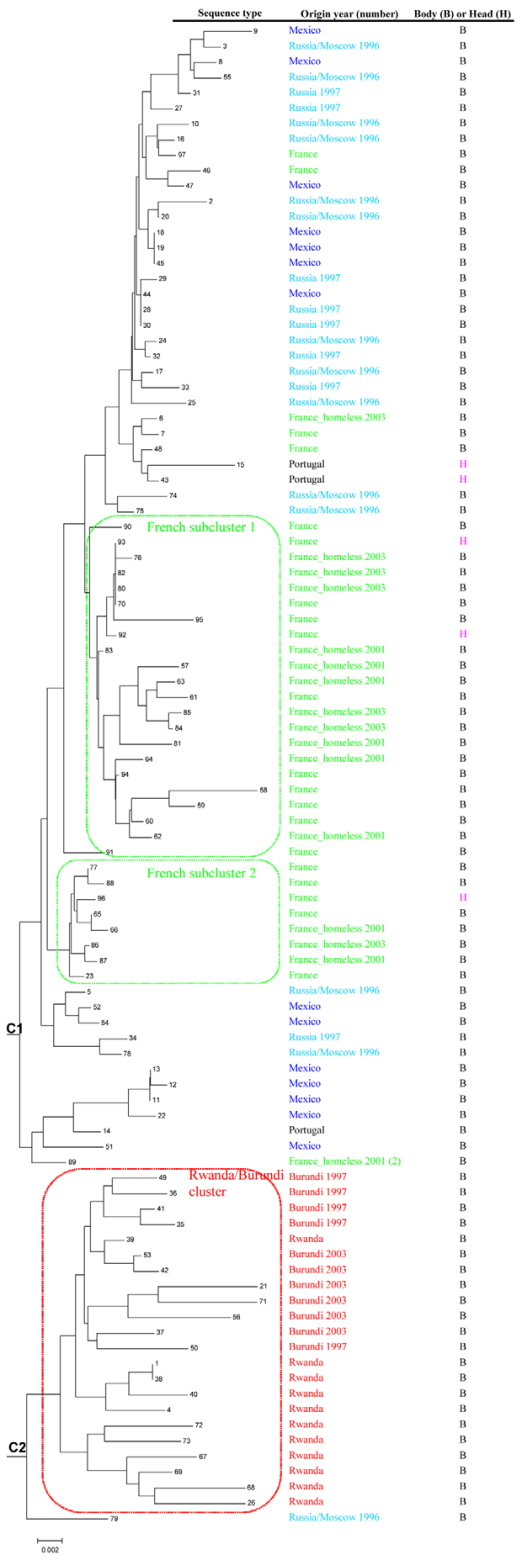
Phylogenetic organization of 97 human lice based on concatenation of four nuclear intergenic spacers, PM1, PM2, S2, and S5, using the neighbor-joining method.

Two hundred and eighty-four human lice collected from Russia, France, Portugal, Mexico, USA, UK, Morocco, Algeria, Peru, Thailand, Australia, Rwanda, and Burundi were included in this study. Lice were collected by experienced entomologists from patients who had only one type of infestation (head or body) and classified according to the site where they were found. Among them, only 97 lice were tested with four nuclear intergenic spacers, other 110 and 77 lice were tested with two intergenic spacers, respectively, due to limited DNA quantity. The strain information, including origin, the body part where they were removed (body or head), and the year when it was collected are given in Figure 1 and Figures S1 and S3. In addition, to estimate the utility of multi-spacer typing (MST) of louse populations, we also studied two body lice from our laboratory colony (Culpeper strain) per year, collected in 1998, 1999, 2000, 2003, 2004 and 2009. From 1998 to 2009, our louse colony went through 132 generations.

All lice were stored at −20°C until processed further. Before DNA isolation, each louse was rinsed twice in sterile water for 15 minutes and cut lengthwise in half. Then, total genomic DNA of each half louse was extracted using the QIAamp Tissue kit (QIAGEN, Hilden, Germany) as described by the manufacturer. The extracted genomic DNA was stored at −20°C until PCR amplification.

### Selection of nuclear intergenic spacers as typing markers and primer design

The nuclear intergenic spacers were randomly selected from the genomic sequence of *Pediculus humanus humanus* UDSA strain (http://phumanus.vectorbase.org/index.php) and were identified with flanking genes which exhibited >40% sequence identity with homologous genes in the Vectorbase [14],[18]. Primers used for amplification and sequencing of these intergenic spacers were chosen from the flanking genes using the Primer 3.0 software (http://frodo.wi.mit.edu/cgi-bin/primer3/primer3_www.cgi) and are listed in Table 1. All Primers used in this project were obtained from Eurogentec (Seraing, Belgium).

**Table 1:** The name, position on supercontig, size, and primers used for amplification of 22 intergenic spacers.

Name	Start	End	Foward primer (5′-3′)	Reverse primer (5′-3′)	Size (bp)
S1	27999	28435	GACCAACCAACCAGCCAATA	TTCCAGAAGCCTTGTTACCG	437
S2	665433	665920	ATGATGTGCATTGCGAGTGT	AAACTTAACCCGGGCCCTAT	488
S3	44210	44721	GAAAGTGACGACGATGACGA	TCCCAATTTTTGTTTCCCTG	512
S4	76618	77129	ACCCGATAAACCGACAGATG	TTTGCCCATACAGCAATTCA	512
S5	69159	69650	TCCAAATGAAACCCACACTTT	TGGCAGACACTGCTTCCTTA	492
S6	63422	63928	AACAAAACAATTTGACCCGC	GCATCTTTAAATCCGACAATTTT	507
S7	188992	189457	ATGATTGCATCACTCCGTCA	CGTTGAGGAATCTGGCATTT	466
S8	64865	65355	GGATTTGCAAAAAGCGGATA	ATTTGCCGGGTAGGGTACTT	491
S9	292090	292560	AAGTTTTGTGCTCAAAGCG	AATTCGCAGACGTAAAGCGT	471
S10	86299	86794	TCTTTAGCAAAACTTGGTGATGAG	TGCCTGAAGAGCTTCACATT	496
S11	22923	23408	AGGAATTGGATGAATTGCTCA	ATGACTGTGACTTCCAGCCC	486
S12	20812	21327	CCGCTGAACGAATCTTTCTC	TCATCCCTTGTTTTTCCACC	516
S13	155485	155887	TGGTTGTTTTTCACCCATTG	AAACCCGAGCGAATGTTTT	403
S14	228690	229166	TAATACGGAAAAATGCGTCG	CGATCGGAATTGTGAGGTTT	477
S15	279599	280127	CCTGAACAACTTGAAAGAATTGC	GGCAAGCCAAAACACCTAAA	529
S16	41948	42461	GGGGAAATAAAACAAGAGGAGG	CAACCGGGTGACCACATTAT	514
S17	248360	248834	TGATTTAGGTGGATTTCACGG	TTTCCAACGAATTTCGAACC	475
S18	321921	322390	ATCTCTGTTTTCAGTGGCGG	TCTGGTTTACAGGTGTCGAAAA	470
S19	204723	205216	AAAACAAACAGACGTAGAAAGCG	GGGGGTAAACAAAATGGGTAA	494
S20	603	1045	GGATTTTCTGTTTTGCGTTTT	TTGGTTCTGCATGAAATACGTT	443
PM1	538639	538857	GAAATAATATCCAACCTCGTTCA	CATTCTTCCTCATCAAGCTGC	217
PM2	115253	115810	CCGAAGGAGCTGATTCTTTT	CCACAACGAGTGATGTGAGC	437

As the testing of all intergenic spacers on all louse samples was labor intensive, a panel of 16 lice from a wide range of origins was first used to test for the validity and the presence of polymorphisms for each of the intergenic spacers. Subsequently, the intergenic spacers with successful amplification and sequencing from the 16 tested louse samples, were finally used as markers in order to genotype the remaining strains.

### Amplification and sequencing of intergenic spacers

PCR amplification of each intergenic spacer was carried out in a PTC-200 automated thermal cycler (MJ Research, Waltham, MA, USA). 1 µl of each DNA preparation was amplified in a 20 µl reaction mixture containing 10 pM of each primer, 2 mM of each nucleotide (dATP, dCTP, dGTP and dTTP), 4 µl of Phusion HF buffer, 0.2 µl of Phusion polymerase enzyme (Finnzymes, Espoo, Finland) and 12.4 µl of distilled water. The following conditions were used for the amplification: an initial 5 min of denaturation at 95°C, followed by 35 cycles of denaturation for 1 min at 94°C, an annealing time of 30 sec at 56°C, and an extension cycle for 1 min at 72°C. The amplification was completed by an extension period of 5 min at 72°C.

PCR products were purified, using the MultiScreen PCR filter plate (Millipore, Saint-Quentin en Yvelines, France), as recommended by the manufacturer. PCR products were then sequenced in both directions, with the same primers used for PCR amplification, using BigDye Terminator version 1.1 cycle sequencing ready reaction mix (Applied Biosystems, Foster City, CA). Sequencing products were resolved using an ABI 3100 automated sequencer (Applied Biosystems). Sterile water was used as a negative control in each assay.

### Amplification and sequencing of partial *cytB* gene

In order to compare the discriminatory power of intergenic spacers with genes, as well as to compare their phylogenetic organizations, the mitochondrial gene, *cytB* (cytochrome b) was amplified and sequenced from those louse samples when there was DNA to perform the PCR experiments. The primers used for this experiment were CytbF1 (5′-GAGCGACTGTAATTACTAATC-3′) and CytbR1 (5′-CAA CAA AAT TAT CCG GGT CC-3′) [19].

### Sequence analysis and phylogenetic analysis

Nucleotide sequences were obtained using Sequencher 4.8 (Gene codes Corp, Ann Arbor, MI, USA). The primers used to amplify intergenic spacers were selected based on flanking gene sequences. The sequences from the coding sequence fragments were not used in the analyses. For each intergenic spacer, and *cytB*, a genotype was defined as a sequence exhibiting a unique mutation. Each genotype was confirmed to be unique by BLASTn search in all the obtained sequences [20]. Multiple sequence alignments were carried out using the Clustal W software [21]. Phylogenetic analysis of the lice that were studied was obtained using the neighbor-joining and maximum parsimony methods within the MEGA 3.1 software with complete deletion [22] and using the maximum likelihood method in PhyML 3.0 with SH-like approximate likelihood-ratio test and HKY85 substitution model [23],[24]. For this purpose, sequences of the selected intergenic spacers were concatenated.

### Comparison of discriminatory power intergenic spacers with *cytB*

The discriminatory power (D) of each intergenic spacer, and *cytB*, was calculated with the Hunter and Gaston's formula [25].
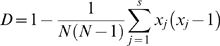



### Accession numbers

DNA sequences obtained from the S2 and S5 spacers, the PM1 and PM2 spacers and the *cytB* gene were deposited in GenBank under accession numbers EU928781-EU928862, EU913096-EU913223, and GU323324-GU323334respectively.

## Results

### Four nuclear intergenic spacers, S2, S5, PM1, and PM2, were selected as typing markers

Twenty-two nuclear intergenic spacers were initially selected from the genomic sequences and preliminary tested on 16 louse samples (Table 1). However, due to non-specific amplification, or low sequencing quality, 18 intergenic spacers were removed from this study. Finally, four intergenic spacers, hereafter termed S2, S5, PM1, and PM2, were used as typing markers in this study (Table 1).

Through amplification and sequencing, 165–185 bp of S2 and 156–189 bp of S5 were obtained from 207 louse samples and133–155 bp of PM1 and 323–328 bp of PM2 were obtained from 174 louse samples. Sequences from the different genotypes of the four intergenic spacers have been deposited in the EMBL/GenBank database with access numbers: EU928781-EU928862 for S2 and S5 and EU913096-EU913223 for PM1 and PM2.

### Genotypic distribution of studied lice based on intergenic spacers

Two hundred and seven lice were differentiated into 84 and 49 genotypes based on intergenic spacers S2 and S5, respectively. Concatenation of S2 and S5 sequences differentiated the 207 lice into 148 genotypes.

One hundred and seventy-four lice were differentiated into 25 and 62 genotypes based on intergenic spacers PM1 and PM2, respectively. Concatenation of PM1 and PM2 sequences differentiated the 174 lice into 77 genotypes.

Further concatenation of S2, S5, PM1, and PM2, discriminated a panel of 97 lice into 96 MST genotypes. Except for two lice collected from French homeless people which shared the MST genotype 89, the other 90 lice exhibited unique MST genotypes based on the concatenation of four intergenic spacers.

Sequences from each of the four intergenic spacers S2, S5, PM1, and PM2 were identical among the 12 body lice from our laboratory colony collected over 12 years. The genotypes obtained were: 8, 6, 18, and 39 for S2, S5, PM1 and PM2, respectively.

### Genotypic distribution of lice based on the mitochondrial gene *cytB*

A partial *cytB* gene sequence was amplified and sequenced from 170 lice. A 316 bp fragment was obtained from each louse after sequence correction and assembling. The *cytB* sequences were used to classify the 170 lice into 11 genotypes. The body lice sampled from our laboratory colony over 132 generations exhibited identical *cytB* sequences (genotype 4).

### Discriminatory power of each marker

The discriminatory power (D) of the intergenic spacers, PM1, PM2, S2, and S5 was respectively 0.6988, 0.8406, 0.9677, and 0.8913. The D value of *cytB* was 0.6445. The D value of concatenation of intergenic spacers varied from 0.9123 for concatenation of PM1 and PM2 to 0.9945 for concatenation of S2 and S5. A D value of 0.9998 was reached by combined use of the four intergenic spacers.

### Phylogenetic organization of lice based on intergenic spacers and partial *cytB*

The dendrograms of studied lice inferred by the methods of neighbor-joining, maximum parsimony, and maximum likelihood exhibited similar phylogenetic organizations (Figures 1 – [Fig pntd-0000641-g002] 3, Figures S1 – S6).

**Figure 2 pntd-0000641-g002:**
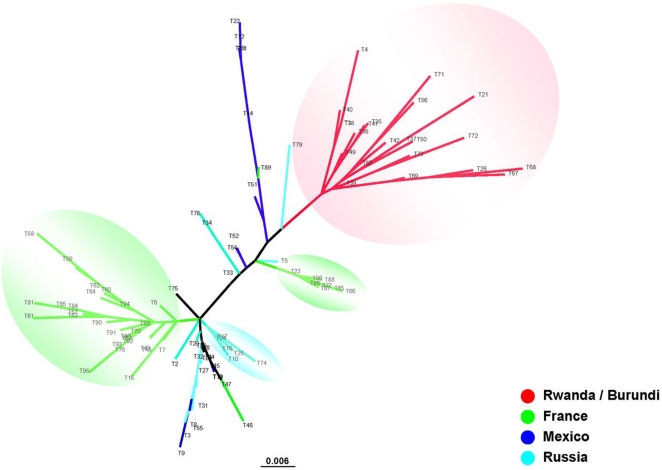
Phylogenetic organization of 97 human lice based on concatenation of four nuclear intergenic spacers, PM1, PM2, S2, and S5, using the maximum likelihood method.

**Figure 3:**
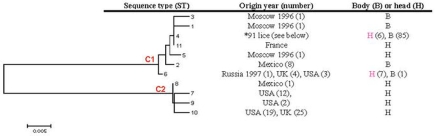
Phylogenetic organization of 170 human lice based on partial *cytB* gene using using the neighbor-joining method.

The 148 genotypes of intergenic spacers S2 and S5, were grouped into 3 clusters, C1, C2, and C3 (Figure S1). Each cluster included both head and body lice. In addition, genotypes 96 and 101 consisted of two body lice and two head lice, respectively (Figure S1). A subcluster (Burundi and Rwanda subcluster) in cluster C3 was comprised of 29 lice from Burundi and Rwanda and one louse from Russia. The other 24 lice collected in Rwanda and Burundi were grouped into cluster C2. The majority of French lice, including those collected from homeless people, were grouped into a sub-clade within cluster C1 (Figure S1).

The 77 nuclear intergenic spacer-genotypes, for PM1 and PM2, were grouped into 2 distinct clusters (Figure S3). Cluster C1 included 148 lice collected from Russia, Mexico, France, UK, USA, and Portugal as well as one louse from Rwanda (Figure S3). A subcluster in cluster C1 contained 31 lice from France and 2 lice from Portugal, which is hereafter referred to as the “French subcluster”. The 19 lice collected from French homeless individuals were tightly grouped with French lice in cluster C1 (Figure S3). Cluster C2 was comprised of 25 lice collected from Rwanda and Burundi. Genotypes 6, 29, and 32, were observed in both head and body lice.

Based on the concatenation of the four intergenic spacers PM1, PM2, S2, and S5, the 97 lice were discriminated into 96 MST genotypes and grouped into two clusters (Figures 1 and 2). Cluster C1 included 75 lice from Russia, France, Mexico, and Portugal, and Cluster C2, the Burundi and Rwanda cluster, contained 22 lice from Burundi and Rwanda (Figure 1). Lice collected from the French homeless individuals were tightly grouped with French lice into two subclusters within C1 (Figure 1).

The 11 genotypes of *cytB* from 170 lice were grouped into 2 clusters (Figure 3). Cluster C1 included 111 lice from France, Russia, UK, USA, Mexico, Portugal, Burundi, and Rwanda (Figure 3), and corresponded to Type A in a study by Light *et al* [5]. Cluster C2 included 59 lice from the UK, USA, and Mexico, and corresponded to the Type B reported by Light *et al.* [5]. Genotypes 4 and 6 comprised both head and body lice.

## Discussion

In this study, MST based on four highly variable intergenic spacers selected from the genomic sequence of a body louse, classified 97 lice into 96 MST genotypes. To date, MST appears to be the most sensitive discriminatory genotyping system of human lice, allowing for discrimination of individuals. In addition, MST helped us to address several important debates associated with human lice. One of the ongoing debates is whether head and body lice are separate species or two subspecies within *Pediculus humanus* [3],[4],[7],[8],[26]. To address this issue, most of the previous studies have used mitochrondrial or nuclear genes to evaluate and compare the genetic variability of human head and body lice. Studies based on the mitochondrial gene *COI* [26], the mitochondrial genes *cytB* and *ND4* and nuclear genes *EF-1α* and *RPII* [27], the mitochondrial genes *cytB* and *COI* [28], or the nuclear gene 18S rDNA [4], supported the hypothesis that human head and body lice are conspecific. Using previously published sequence data, by reticulated networks, gene flow, population genetics, and phylogeny analysis, Light *et al*. [8] also observed that human head and body lice are conspecific.

However, a recent study performed by Leo *et al*. [7], in which microsatellites were used as genetic markers, concluded that human head and body lice are two distinct species. These studies made opposite conclusions by using different genetic markers. The low discriminatory power of previously used markers limited their ability to provide convincing evidence whether head and body lice are subspecies of one species or two distinct species.

Our genotypic and phylogenetic analyses using MST did not support the hypothesis that human head and body lice are separate species. For instance, genotype 32, which was a concatenation of the intergenic spacers PM1 and PM2, was comprised of 44 head lice from the USA and the UK as well as one body louse from Europe (Figure S3). Genotypes 6 and 29 were comprised of both head and body lice collected in France (Figure S3). Genotypes 96 and 101, a concatenation of the intergenic spacers S2 and S5, also were comprised of both head and body lice (Figure S1).

Phylogenetic organizations of head and body lice based on each of the four intergenic spacers, and on concatenation of both, support the hypothesis that head lice were grouped with body lice in the same clusters or subclusters (Figure 1, Figures S1 and S2). The changing tree topography observed among spacers may be related to differences in selection pressure that their flanking genes undergo. The genotypic distribution of 170 lice based on partial *cytB* gene sequences, and the phylogenetic organization of 11 *cytB* genotypes, also demonstrated that head and body lice shared the same *cytB* genotypes and were grouped in the same cluster (Figure 2), which further confirmed the hypothesis that human head and body lice are conspecific [3],[27],[28].

In our study, although only two clusters were observed based on partial *cytB* gene sequences, cluster C1 contained both head and body lice from worldwide origins, and cluster C2 included only head lice from America and Europe (Figure 3). This result did not contradict the previous observation [19] of three deeply divergent clades of human lice, as our study did not include lice from either Ethiopia or Nepal. However, the phylogenetic organization of *cytB* sequences was significantly simpler than those based on intergenic spacers. Three and two clusters were respectively obtained from the concatenations of the intergenic spacers S2 and S5 (Figure S1), and the intergenic spacers PM1 and PM2 (Figure S3). Additionally, two clusters were generated from the concatenation of the four intergenic spacers PM1, PM2, S2 and S5 (Figure 1, Figures S1 and S2). Each cluster was comprised of several subclusters, such as the French subcluster, including the majority of French lice, and the Rwanda/Burundi cluster, which also consisted of lice collected from sub-Saharan Africa (Figure 1, Figures S1 and S2). This discrepancy of phylogenetic organizations obtained from intergenic spacers and *cytB* sequences resulted, at least partially, from the high variability of intergenic spacers, which enabled individual discrimination of human lice. In addition, these differences may also be explained by the fact that the louse samples incorporated in each phylogenetic analysis were different due to limited DNA available for such experiments. Furthermore, louse genomic DNA may be highly recombined, which would in turn result in distinct phylogenetic organization from different markers [8]. Thus, collecting more louse samples with wide origins, especially lice from Ethiopia and Nepal, and subjecting them to MST analysis, would help to further clarify the relationship between head and body lice.

Human head and body lice are strict obligate human ectoparasites that differ in several aspects of their morphology, physiology and life histories. Head lice are mostly found on the head and attach their eggs to the base of hair shafts, whereas body lice reside in clothing and attach their eggs to clothing fiber, a life history strategy that probably arose when humans first began wearing clothes [27]. By comparison with body lice, head lice have been described as having shorter and broader antennae, shorter legs, more marked indentations between successive abdominal plates, and as being larger and more deeply pigmented [29],[30]. However, such morphological differences have been determined on a small number of lice and may not hold at the species level [29]. Body lice also take a larger blood meal, lay higher numbers of eggs and develop faster than head lice [29],[31],[32]. In addition, body lice are more resistant to environmental conditions, can stay alive for longer period of time outside the host, are able of transmitting infectious diseases, and are mostly found in adults whereas head lice are essentially found in children [29]. Despite various genetic differences [1]–[8], detailed above, head and body lice have been shown to be able to interbreed [30].

Lice are extremely well adapted ectoparasites, which are usually host-specific by co-speciation with their host [2],[28],[33]. Thus, lice have become a good genetic model for studying specific aspects of human evolution, including addressing when our human ancestors began to wear clothing. Very recently, a study based on sequence analysis of *COI* and *cytB* from human head and body lice suggested direct contact between modern and archaic humans [28]. More recently, Light *et al.* [34] verified this hypothesis by using both nuclear and mitochondrial genes [34]. However, these studies were based on conserved mitochondrial or nuclear genes, which provided limited genetic variability of studied lice. In our study, we also tested the use of highly variable intergenic spacers for strain-typing of human lice to explore human evolutionary history. Concatenation of these highly variable intergenic spacer sequences classified some lice from Rwanda and Burundi into a basal cluster or subcluster and grouped other lice collected in Rwanda and Burundi with lice from North Africa, Europe, USA, and Asia, which supports the hypothesis that human beings originated in Africa (Figure 1, Figures S1 and S2). Thus, highly variable intergenic spacer sequences could be used to study the evolution history of human lice and its host. It might be argued that, due to fast evolution and high polymorphism, intergenic spacers may not be able to fully reflect long-term dynamic changes of populations. However, we observed that MST was a valuable tool for tracing distinct louse populations, and was not biased by mutations that might arise within a single population over time, for at least 132 generations. Nevertheless, we recommend using a combination of coding genes and intergenic spacers because coding genes are conserved enough to highlight evolutionary relationships, and the intergenic spacers are variable enough to identify fine-scale genetic variability.

While lice may present a valuable model to study its host evolution, human head and body lice cause serious health and social problems. Head lice are common worldwide, infesting millions of school children every year and the resistance of *Pediculus humanus capitis* to insecticides is spreading [35]. Body lice are less prevalent parasites, associated mainly with those living in poor conditions, but are potentially more harmful because they are known vectors of at least three bacterial pathogens in humans: *R. prowazekii*, *B. quintana*, and *B. recurrentis*. There have been several outbreaks of louse-borne *R. prowazekii* infections in Burundi and Rwanda jails in 1997 and 2001, and sporadic *R. prowazekii* infections were also recently reported [9]. Epidemiological surveys of these louse-borne diseases are also very important for us to understand and potentially combat these diseases. In addition, recent evidence has been brought that head lice are potential vectors of *B. quintana* [36],[37], and their role in the epidemiology of epidemic typhus has been questioned [38]. Other studies have identified two endosymbiotic bacteria that have co-evolved in head and body lice [39]–[41]. However, whether these symbionts have any influence on louse behavior, development and/or competence as disease vectors is as yet mostly unknown.

Based on phylogenetic analysis of the four intergenic spacers, S2, S5, PM1, and PM2 as well as a concatenation of them, the head and body lice collected from Rwanda and Burundi tightly grouped together to form clusters as well as subclusters (Figure 1, Figures S1 and S2). In addition, the lice collected from homeless people in France grouped tightly with those collected in non-homeless French people, which suggested louse populations migrate between homeless people and non-homeless people in France and homeless people are known to be at high risk for louse-borne diseases [9],[11],[12].

MST may ultimately be a good tool for performing surveys associated with louse transmission and louse-borne diseases. In addition, our MST analysis demonstrated that head and body lice collected in Rwanda and Burundi in 1997, 2001, 2003, and 2008, were closely grouped (Figure 1, Figures S1 and S2). Thus, the outbreak of louse-borne *R. prowazekii* infection that happened in 1997 and 2001 opens up the possibility that lice in this region may still pose a risk for the transmission of *R. prowazekii* to humans. However, clear separation of African lice (collected from Rwanda and Burundi) from other lice was not recovered by intergenic spacers S2 and S5, likely due to recent recombination of nuclear DNA [8].

**Figure 4:**
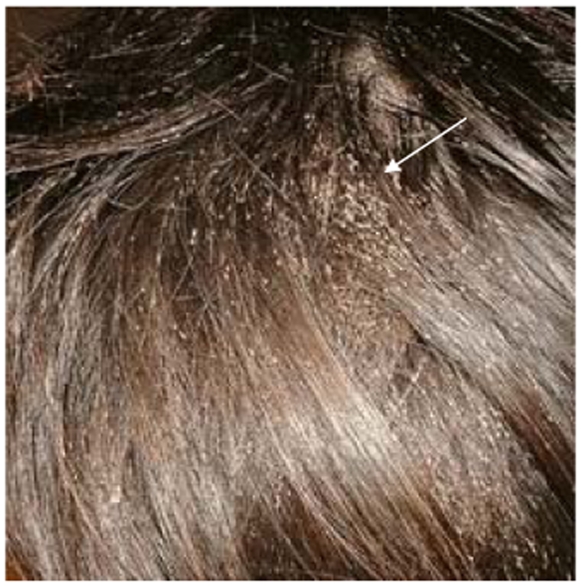
Heavily infested hair with *Pediculus humanus capitis* (arrow). The patient gave informed consent for use of this picture.

**Figure 5:**
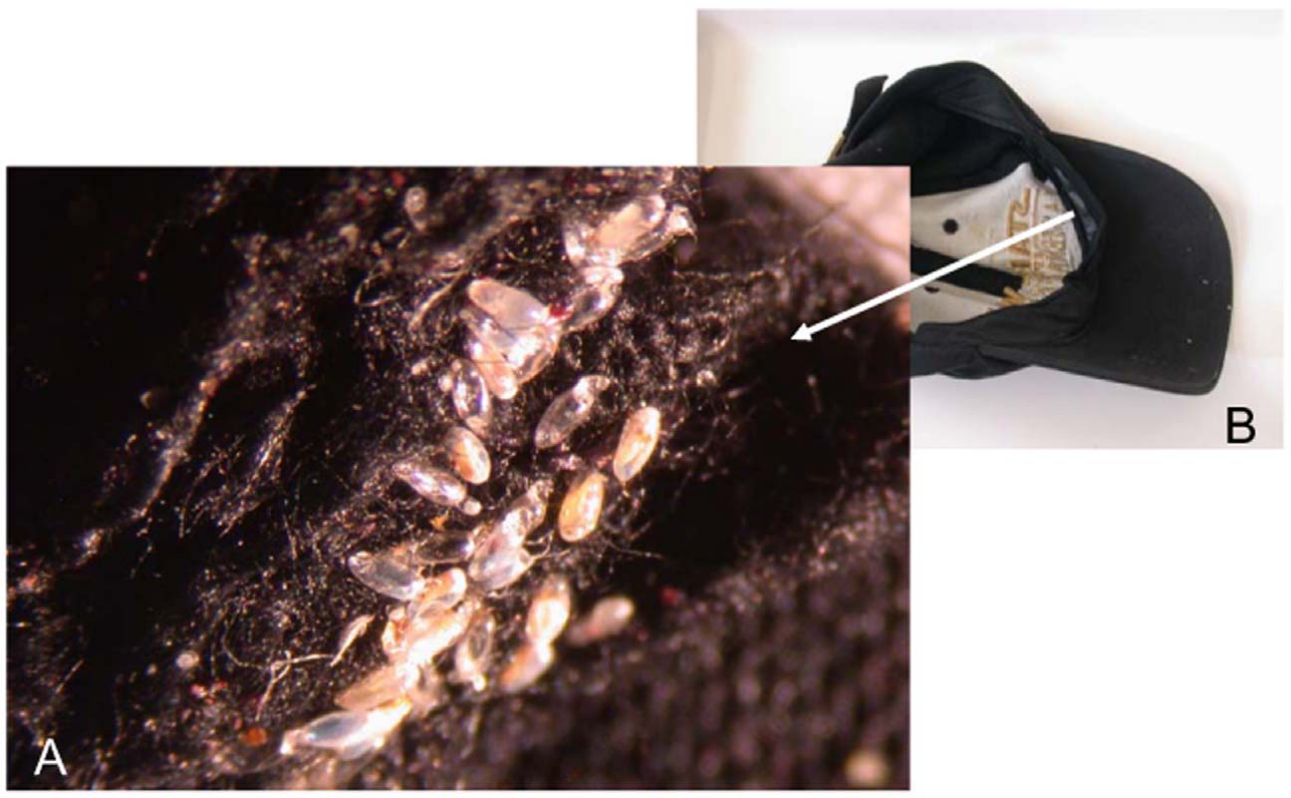
*Pediculus humanus capitis* eggs attached to tissues (A) in a cap (B). The patient gave informed consent for use of this picture.

**Figure 6:**
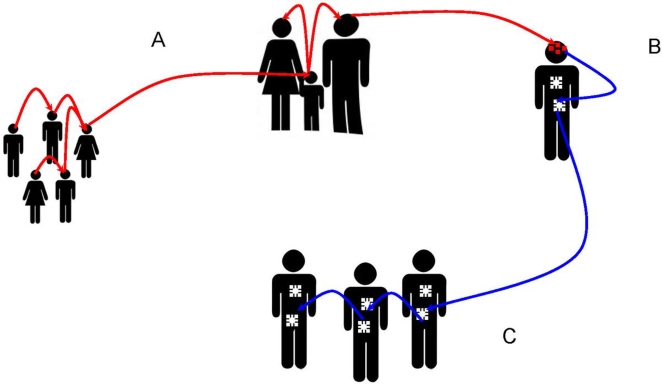
Proposed scenario for the evolution of head and body lice. (A) Head lice propagate among humans; (B) in heavily infested patients, head lice might lay eggs in clothes, and resulting lice might develop and subsequently spread among humans as body lice.

As mentioned above, head lice are different from body lice morphologically and physiologically. It is possible that these phenotypic differences are controlled by a single mutation or potentially a regulatory gene (or genes) governing, for example, the volume of ingested blood. This is the simplest explanation to understand the genetic data showing that lice have exactly the same origin. Under certain conditions of low hygiene, a head louse infestation can transform into a massive infestation (Figure 4). Certain head lice could colonize clothing (Figure 5), and produce a body louse variant by purifying selection or allotropism, which can in turn generate an epidemic of body lice (Figure 6). Several previous observational studies had also suggested that head lice could become body lice when raised in appropriate conditions [42]–[44]. If this scenario is true, the body louse reservoir is not autonomous and actually depends upon head lice. Previous work has shown that all body lice arose from mitochondrial Type A [5], which suggests that only that genotype has the ability to evolve into the body louse niche. This also makes it possible to understand the difficulties to eradicate body lice in a community, especially when the patients are surrounded by other individuals that are infested by head lice. In our clinical work in Marseilles, France, despite 10 years of attempts to minimize human louse populations, body lice continually reappear and may be due to the persistence of head louse populations [45],[46]. Recent work demonstrating the presence of *B. quintana* in head lice [36],[37] suggested that they might also transmit infectious diseases, which supports our hypothesis presented in Figure 6, giving them a greater opportunity to ingest circulating bacteria [29],[31], and that head lice are rarely collected and tested, even when present, in outbreaks of louse-borne infections, may explain why head lice have long been considered to be free from human pathogens.

In conclusion, by strain-typing of human head and body lice using both coding sequences and highly variable intergenic spacers, our data supports the hypothesis that human head and body lice belong to the same species. Based on genotypic and phylogenetic analyses, we also hypothesize that head lice may transform into body lice [47] and cause outbreaks of louse-borne diseases. However, more efforts on the genetic studies of head and body lice are necessary to link their genetic difference with morphological and physiological diversity. Whole genome sequencing of head lice and comparative genomics between head and body lice would be useful to address these questions. In addition, due to its high resolution and reasonable phylogenetic classification, MST based on highly variable intergenic spacer sequences may be helpful for the epidemiological survey of louse-borne diseases.

## Supporting Information

Figure S1 Phylogenetic organization of 207 human lice based on concatenation of two nuclear intergenic spacers, S2 and S5, using the Neighbor-joining method.
(0.02 MB PDF)

Figure S2 Phylogenetic organization of 207 human lice based on concatenation of two nuclear intergenic spacers, S2 and S5, using the Maximum parsimony method.
(0.02 MB PDF)

Figure S3 Phylogenetic organization of 174 human lice based on concatenation of two nuclear intergenic spacers, PM1 and PM2, using the Neighbor-joining method.
(0.04 MB PDF)

Figure S4 Phylogenetic organization of 174 human lice based on concatenation of two nuclear intergenic spacers, using the Maximum parsimony method.
(0.04 MB PDF)

Figure S5 Phylogenetic organization of 97 human lice based on concatenation of four nuclear intergenic spacers, PM1, PM2, S2, and S5, using the Maximum parsimony method.
(0.05 MB PDF)

Figure S6 Phylogenetic organization of 170 human lice based on partial sequence of the *cytB* gene, using the Maximum parsimony method.
(0.02 MB PDF)
