# Supplementary figures and images for: Genotyping of Human Lice Suggests Multiple Emergences of Body Lice from Local Head Louse Populations
Source: PLoS Negl Trop Dis. 2010 Mar 23;4(3):e641. doi: 10.1371/journal.pntd.0000641 (PMC2843630; doi:10.1371/journal.pntd.0000641)

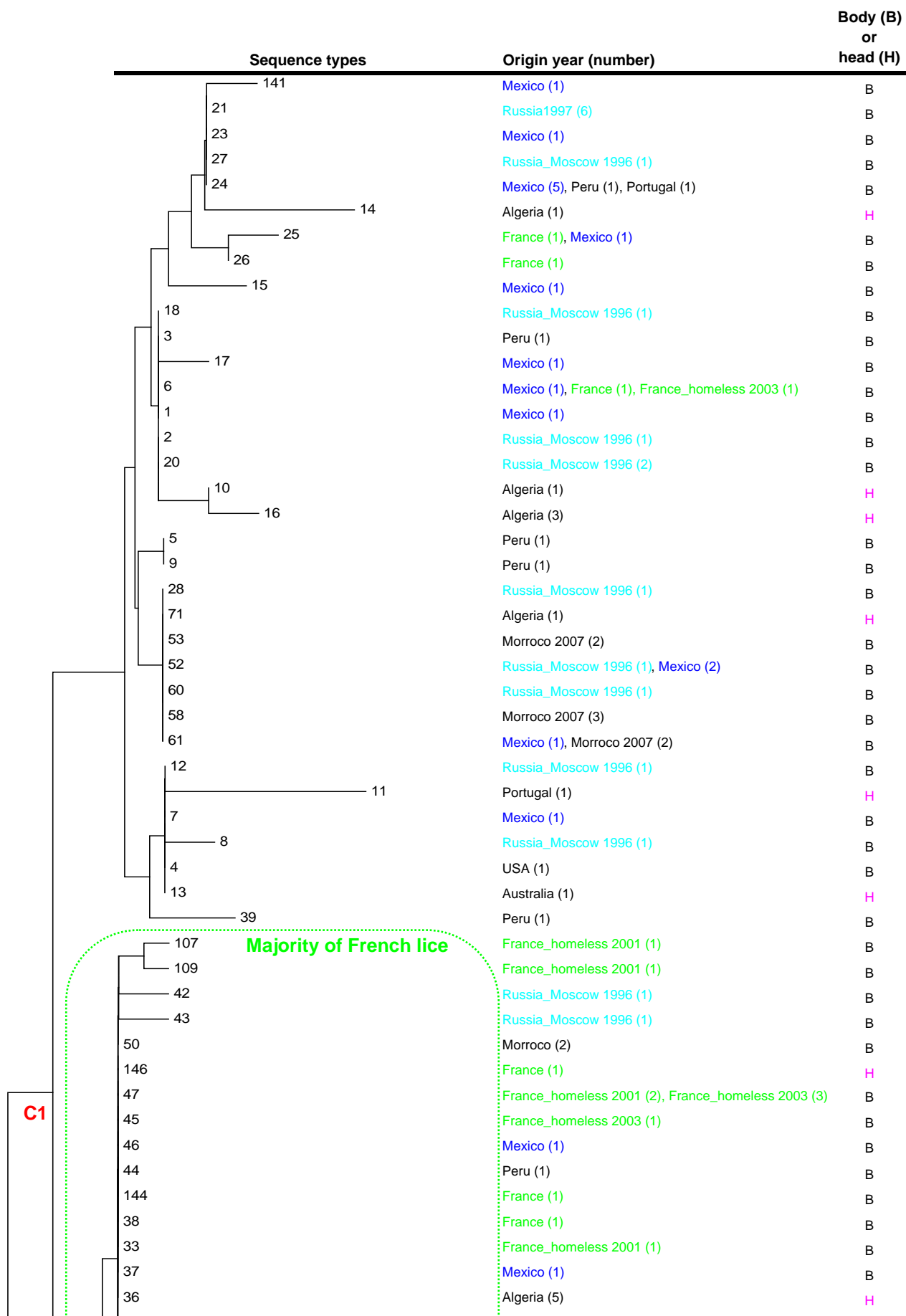

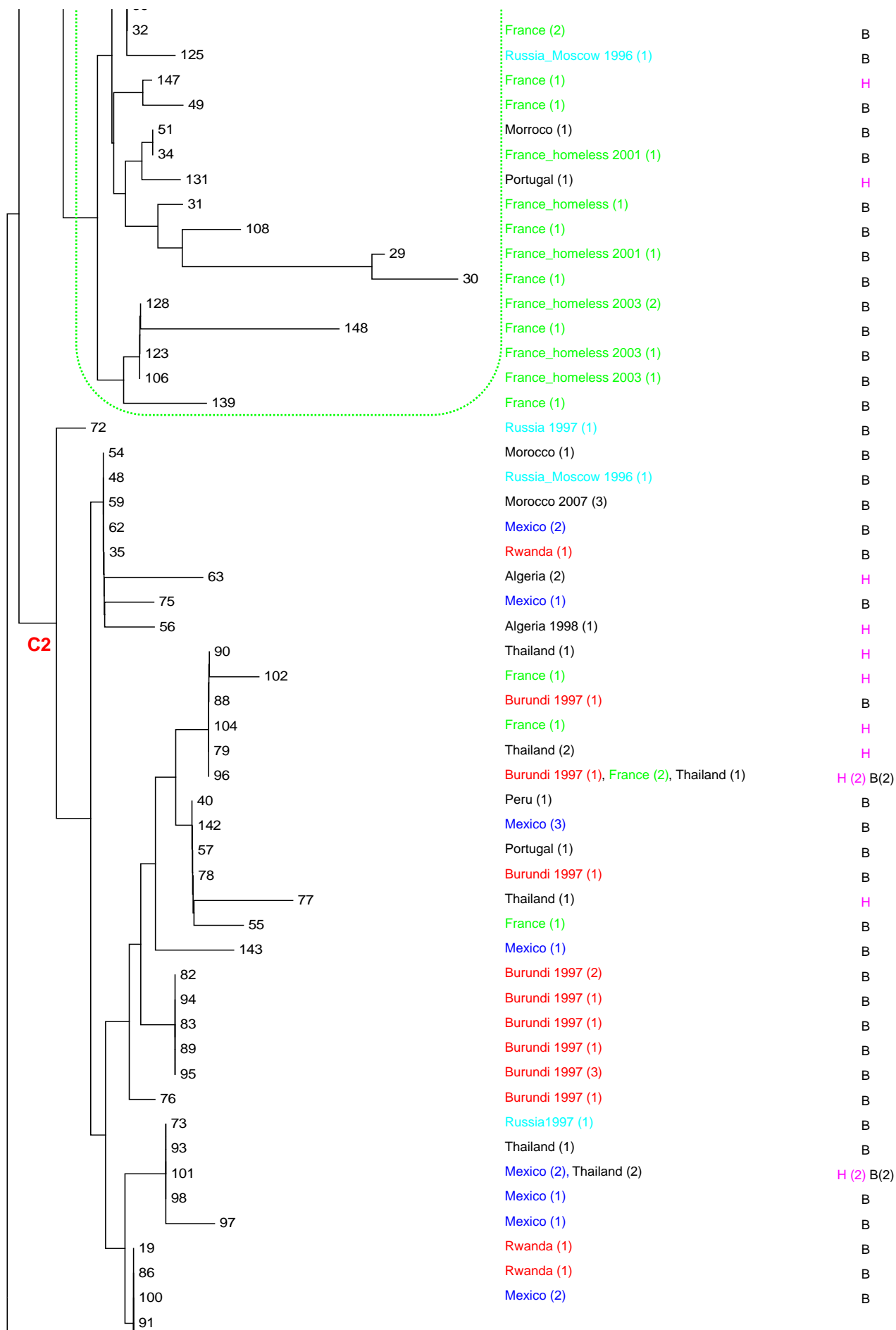

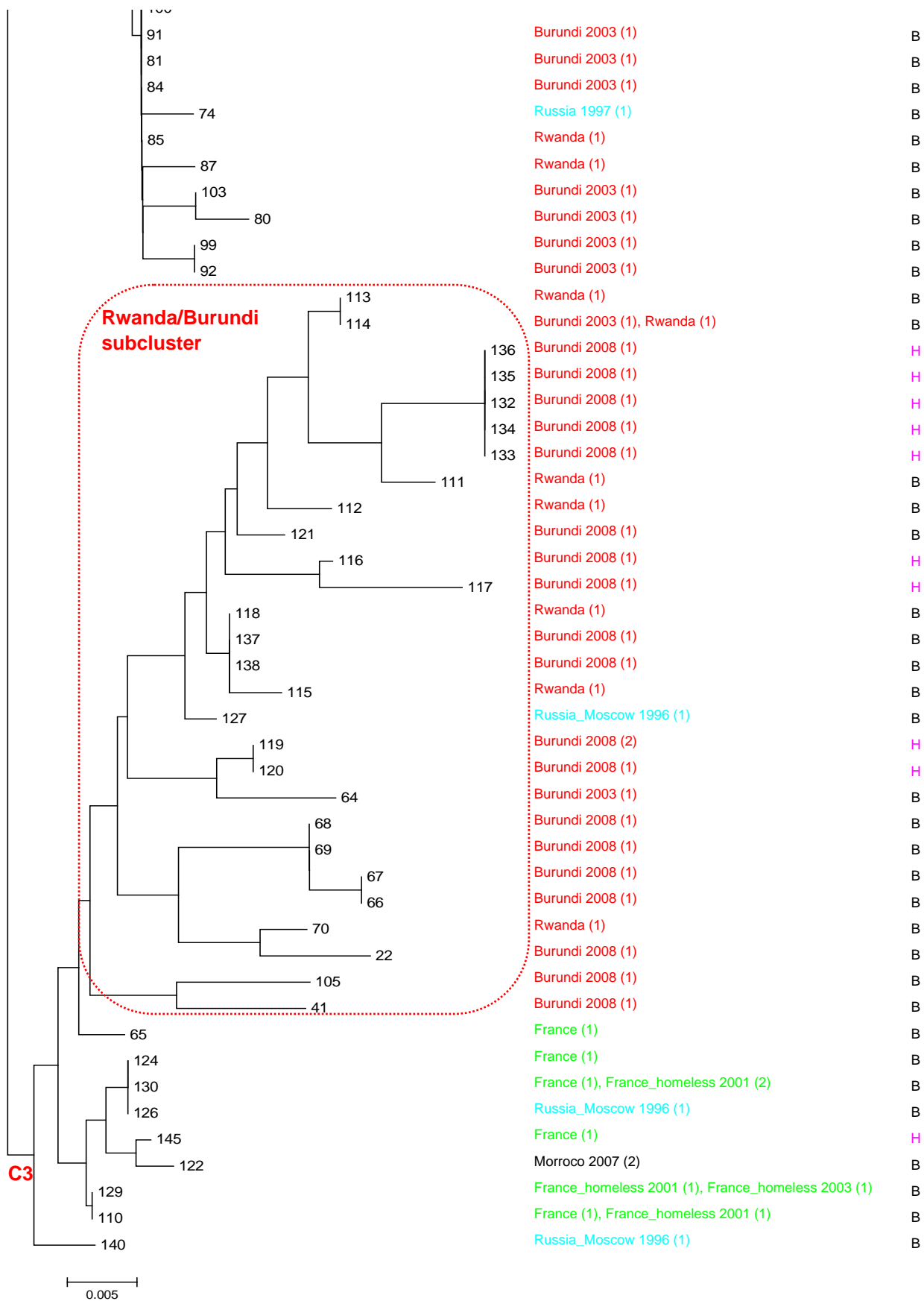

Supplement: Figure S1 — Phylogenetic organization of 207 human lice based on concatenation of two nuclear intergenic spacers, S2 and S5, using the Neighbor-joining method. (0.02 MB PDF) [file pntd.0000641.s001.pdf]

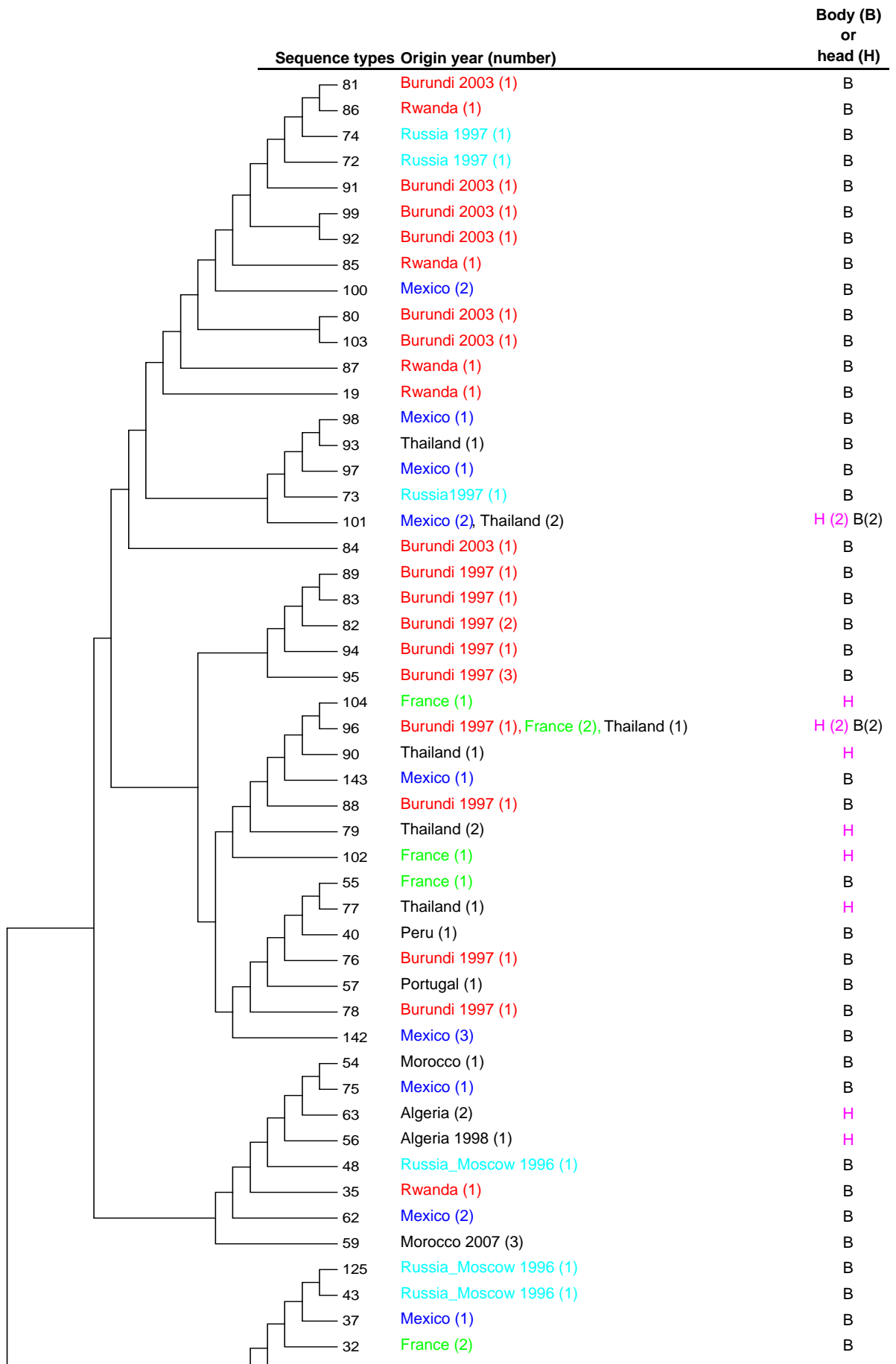

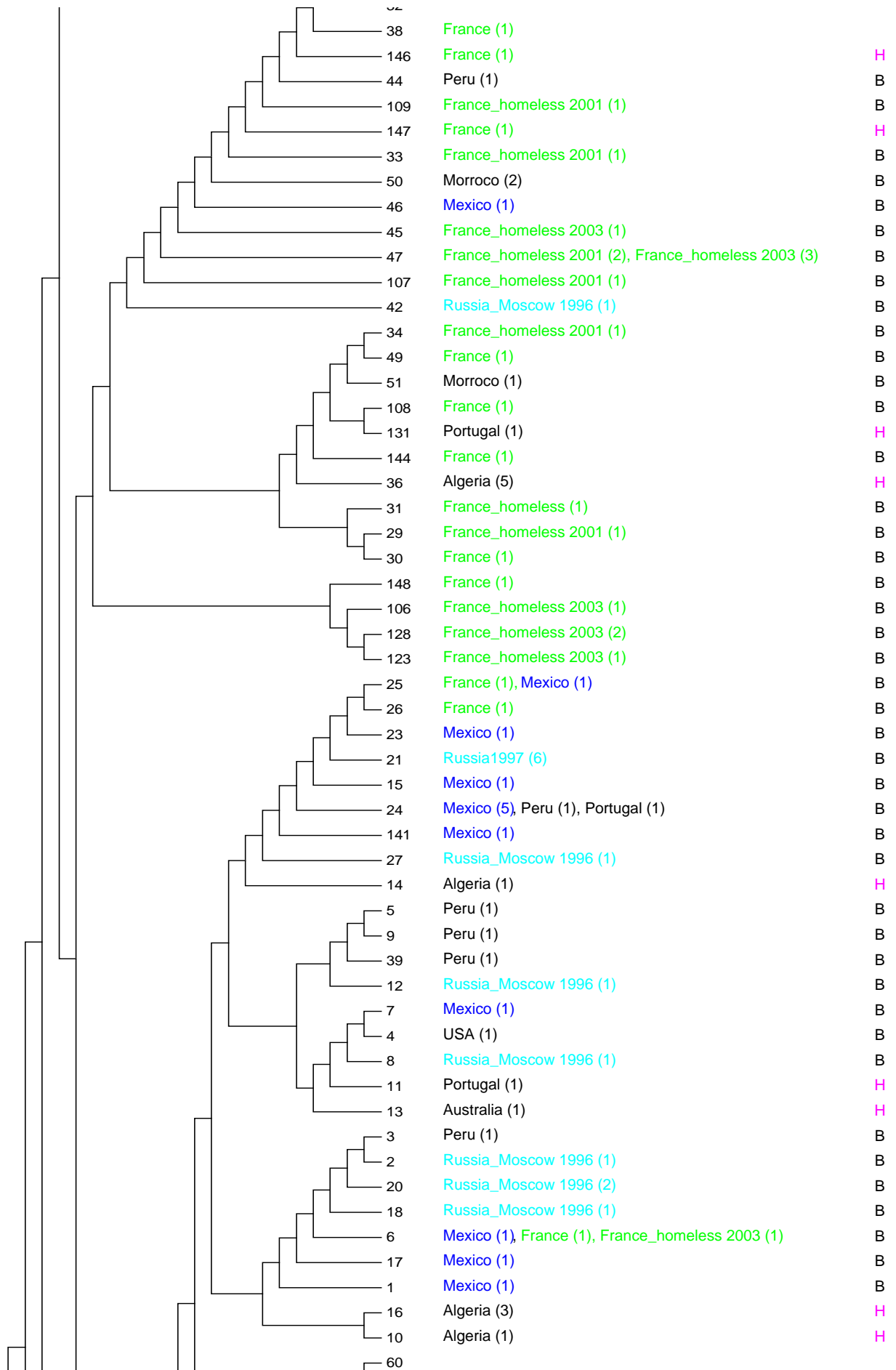

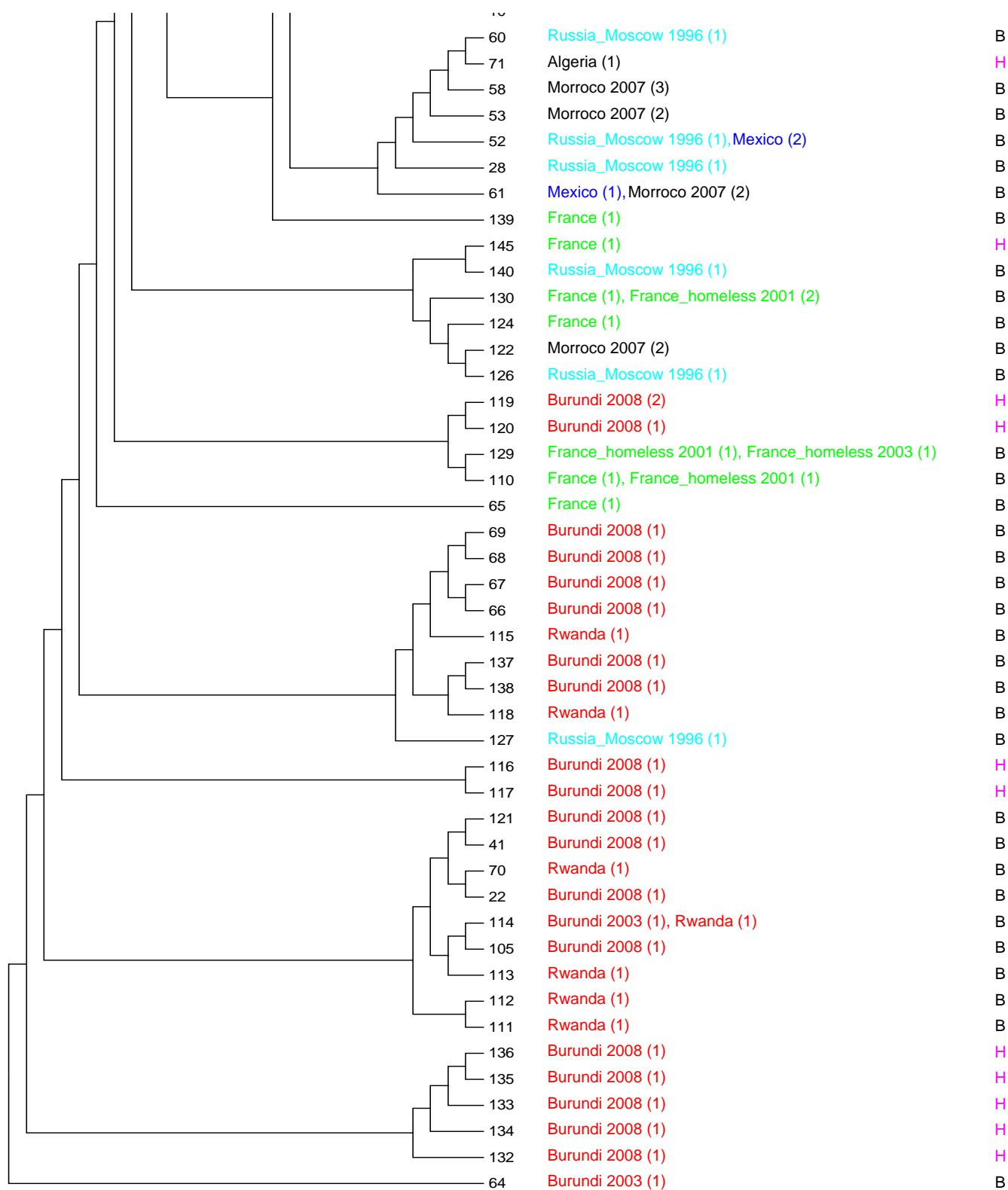

Supplement: Figure S2 — Phylogenetic organization of 207 human lice based on concatenation of two nuclear intergenic spacers, S2 and S5, using the Maximum parsimony method. (0.02 MB PDF) [file pntd.0000641.s002.pdf]

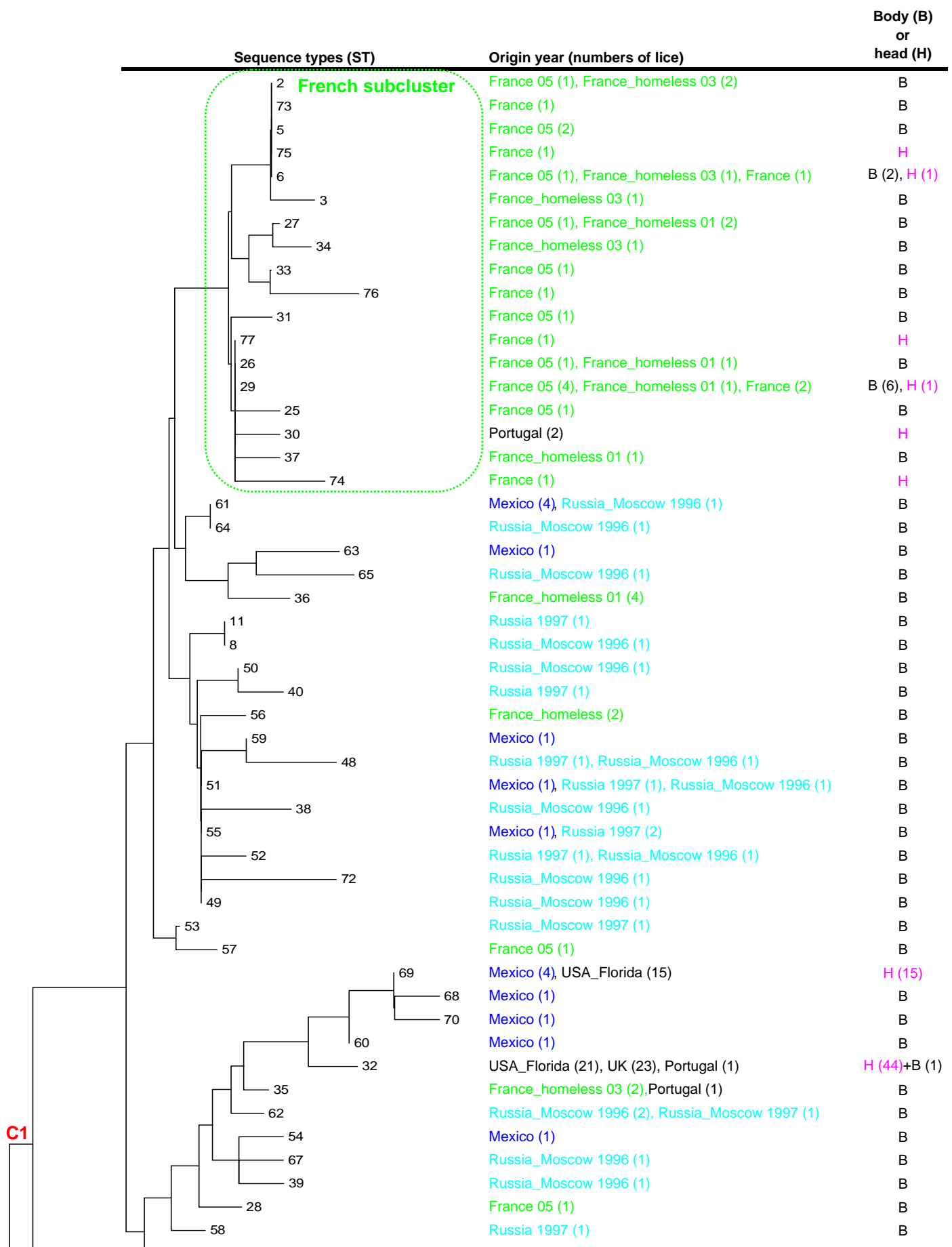

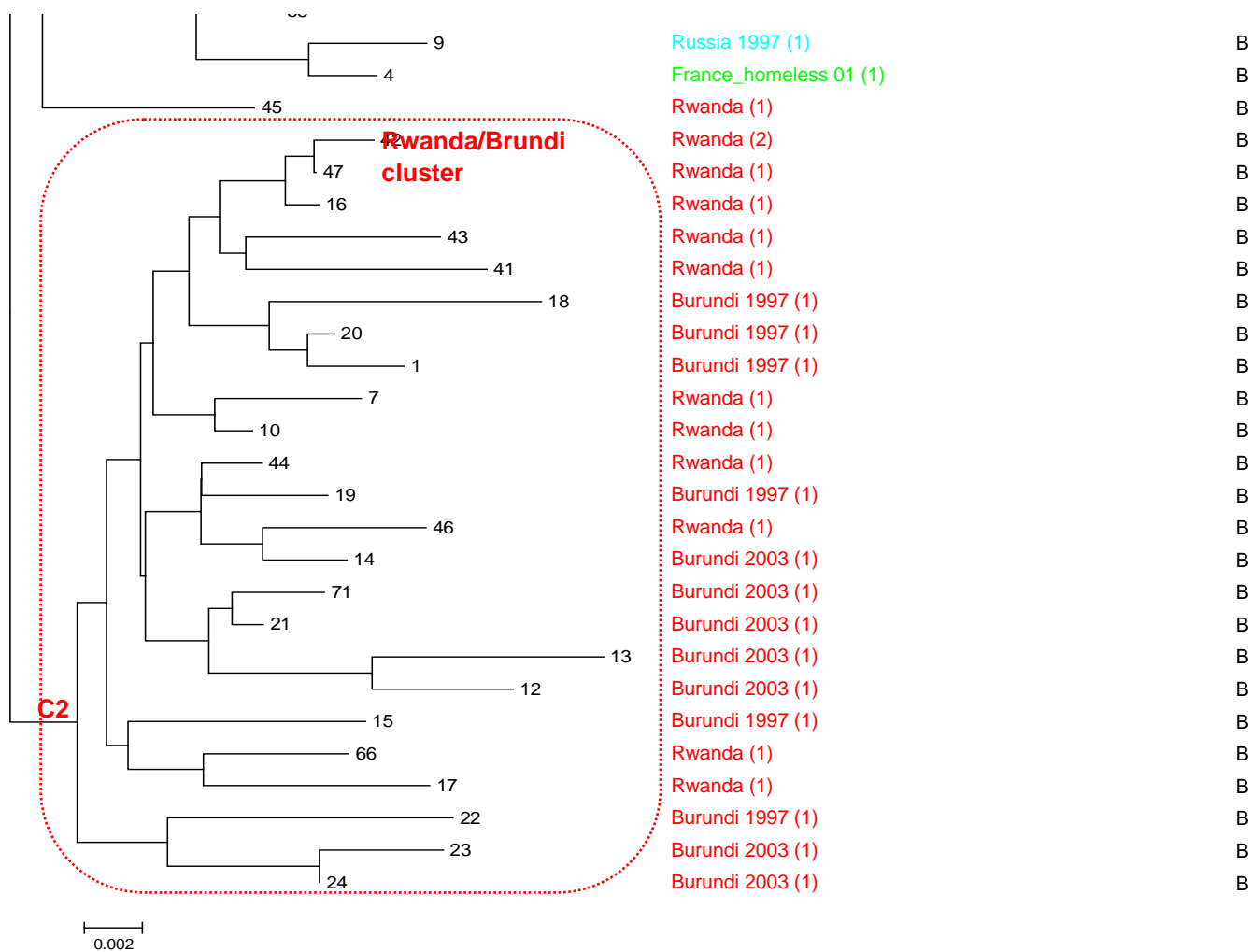

Supplement: Figure S3 — Phylogenetic organization of 174 human lice based on concatenation of two nuclear intergenic spacers, PM1 and PM2, using the Neighbor-joining method. (0.04 MB PDF) [file pntd.0000641.s003.pdf]

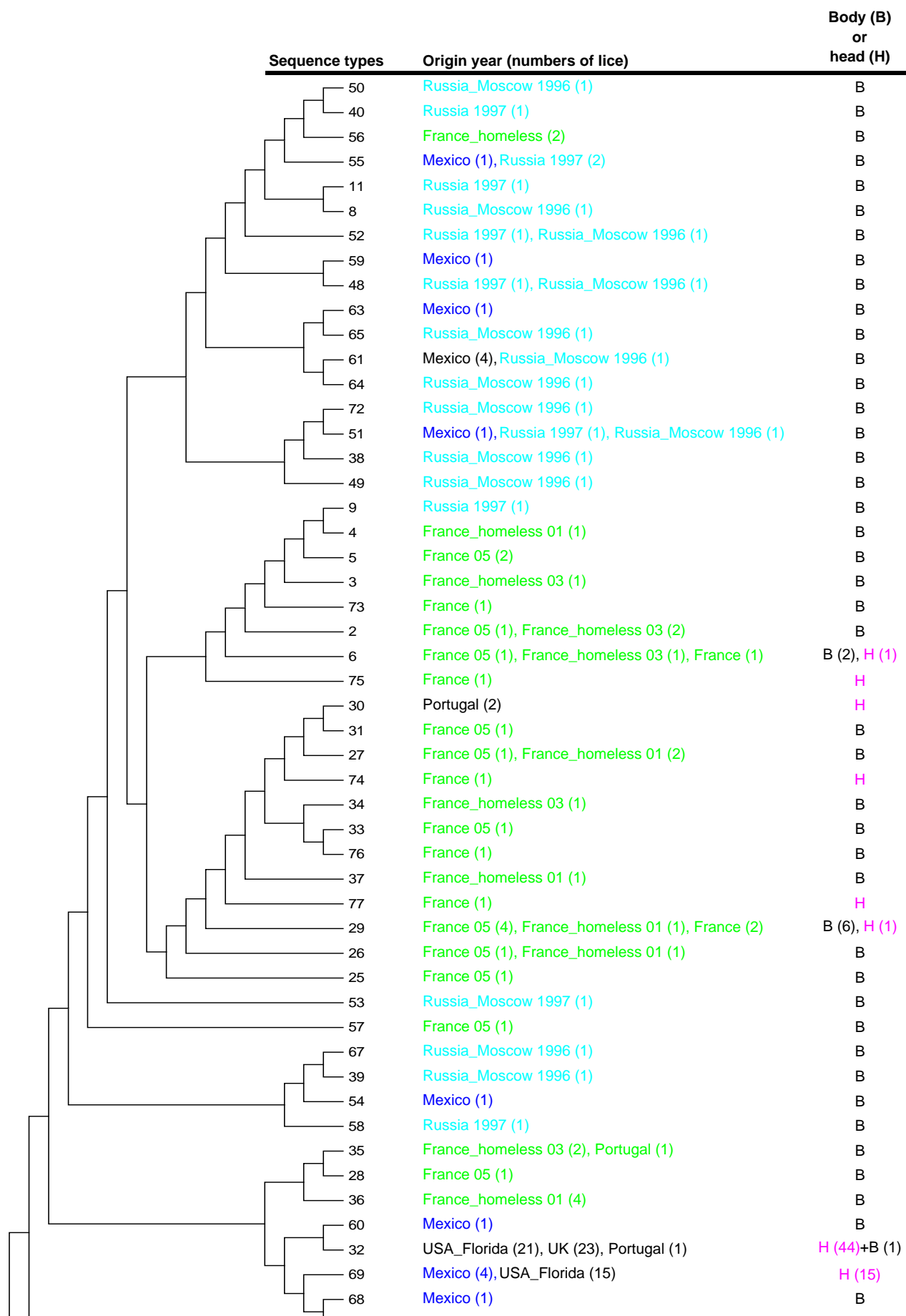

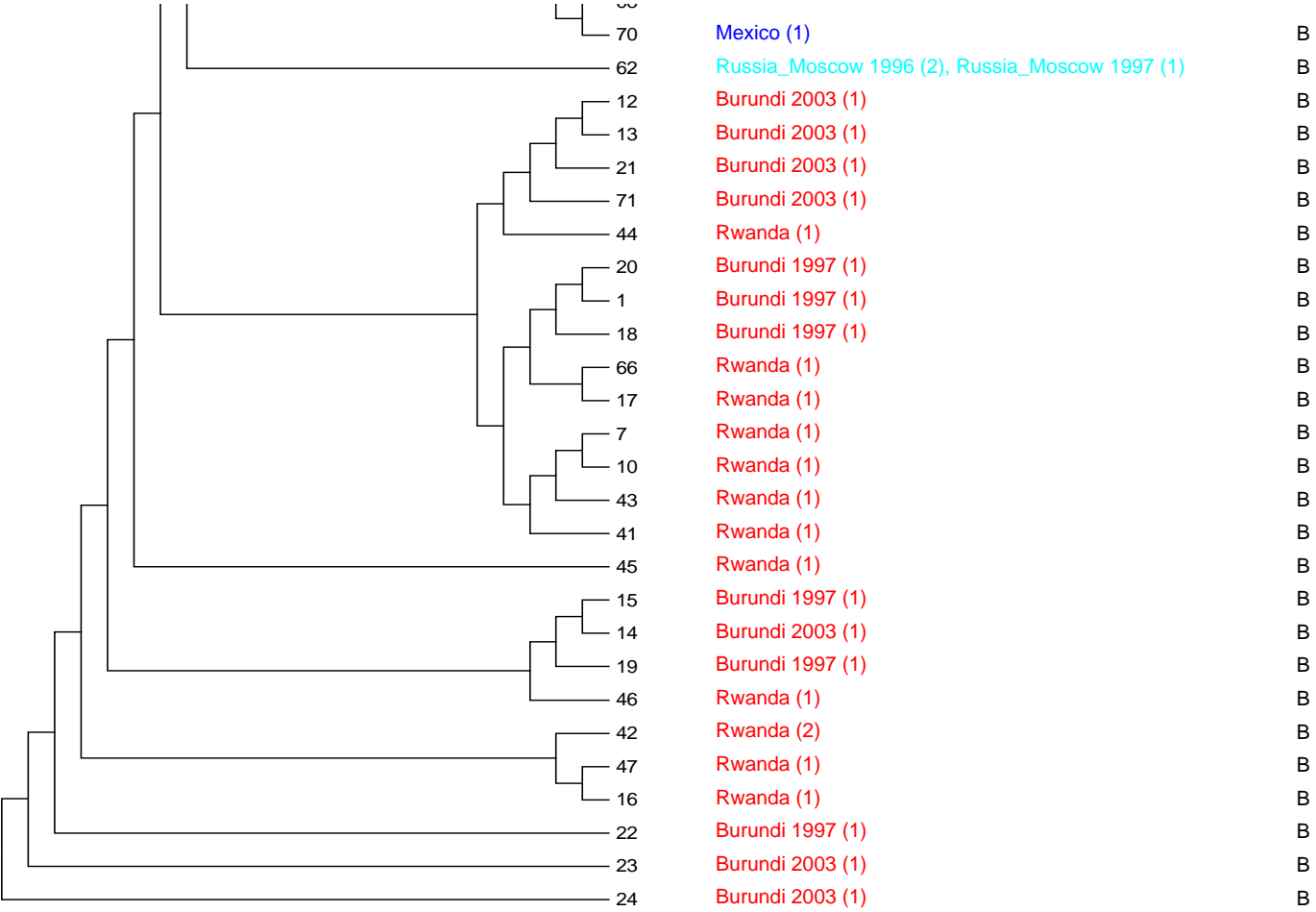

Supplement: Figure S4 — Phylogenetic organization of 174 human lice based on concatenation of two nuclear intergenic spacers, using the Maximum parsimony method. (0.04 MB PDF) [file pntd.0000641.s004.pdf]

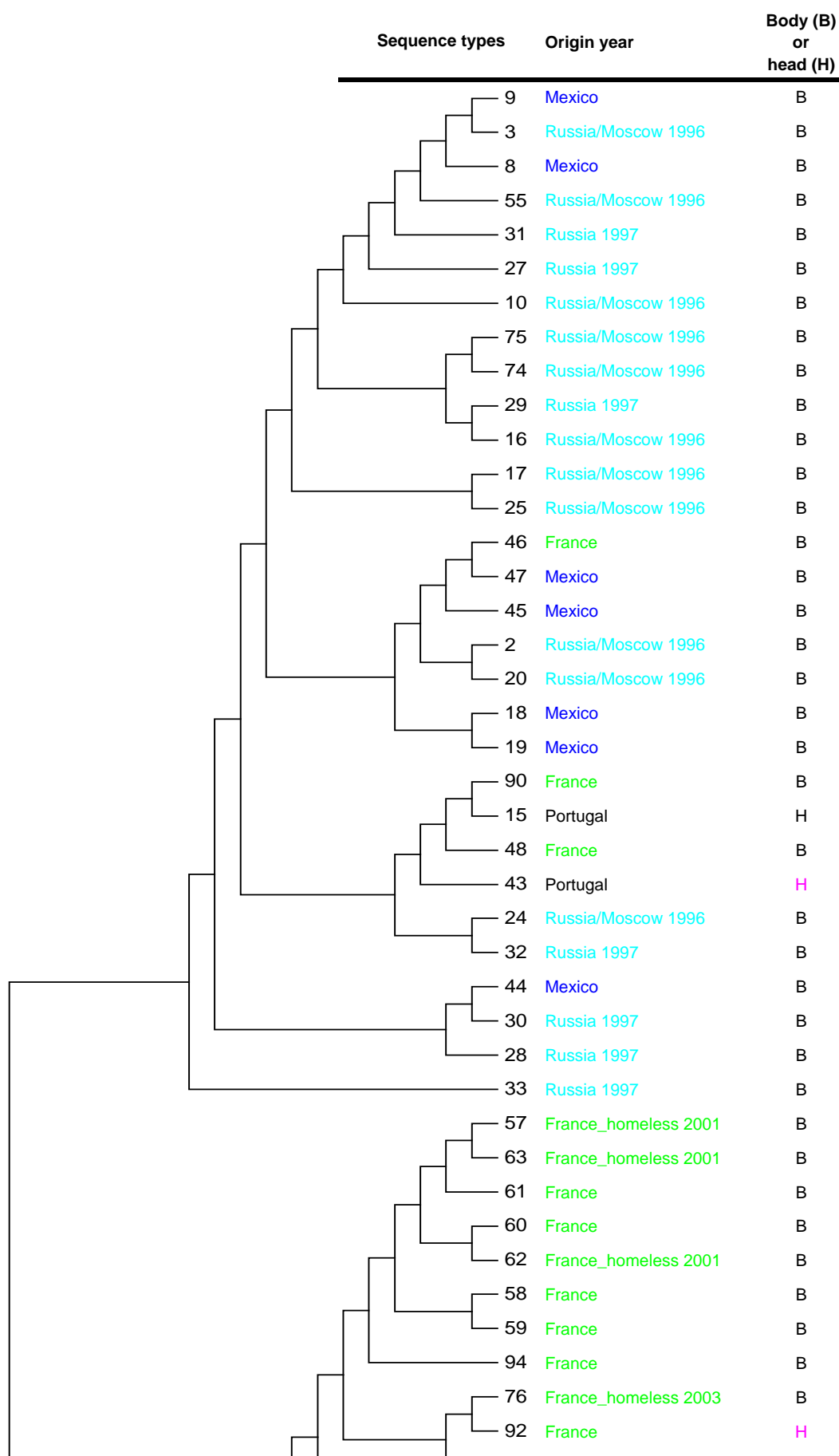

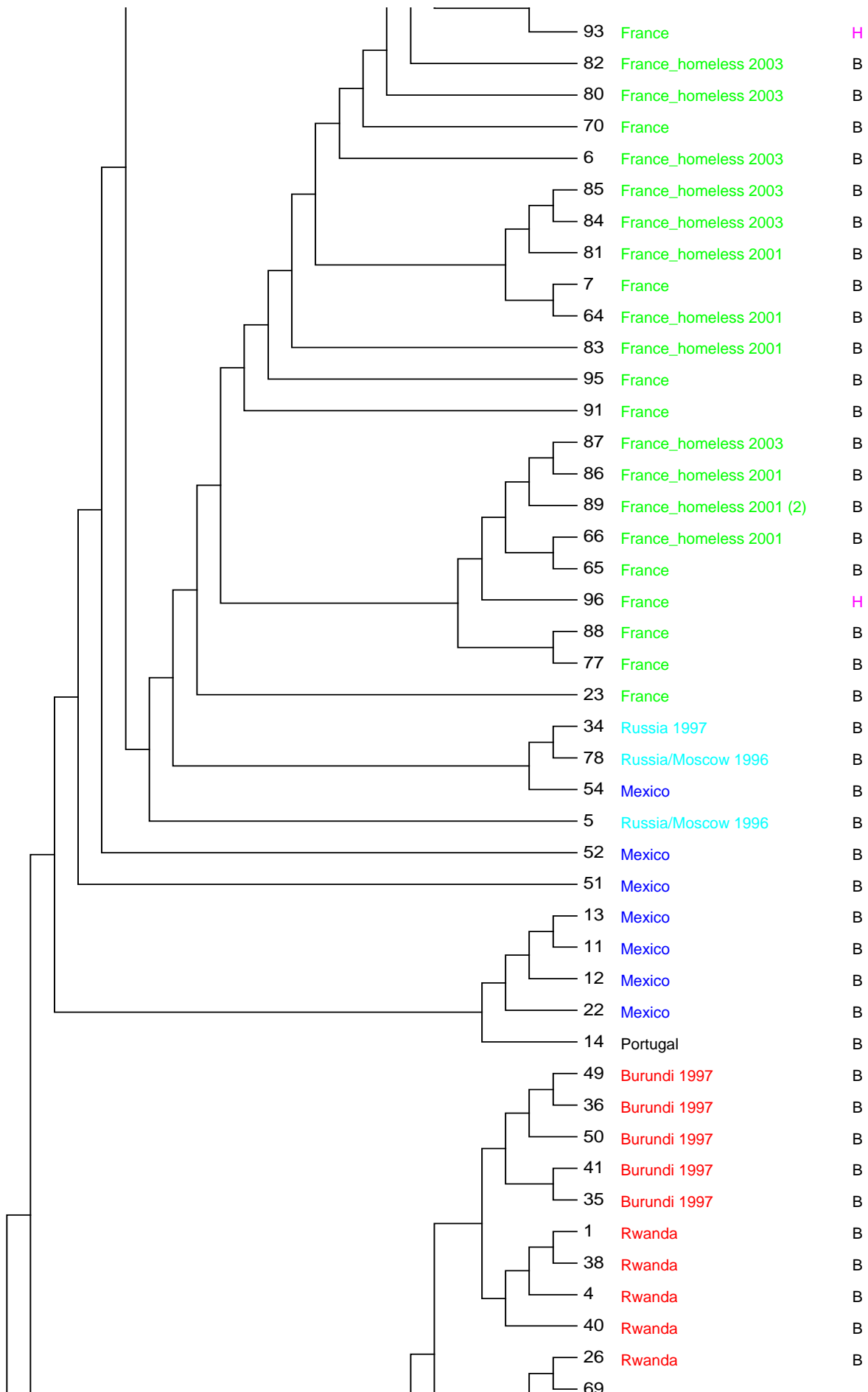

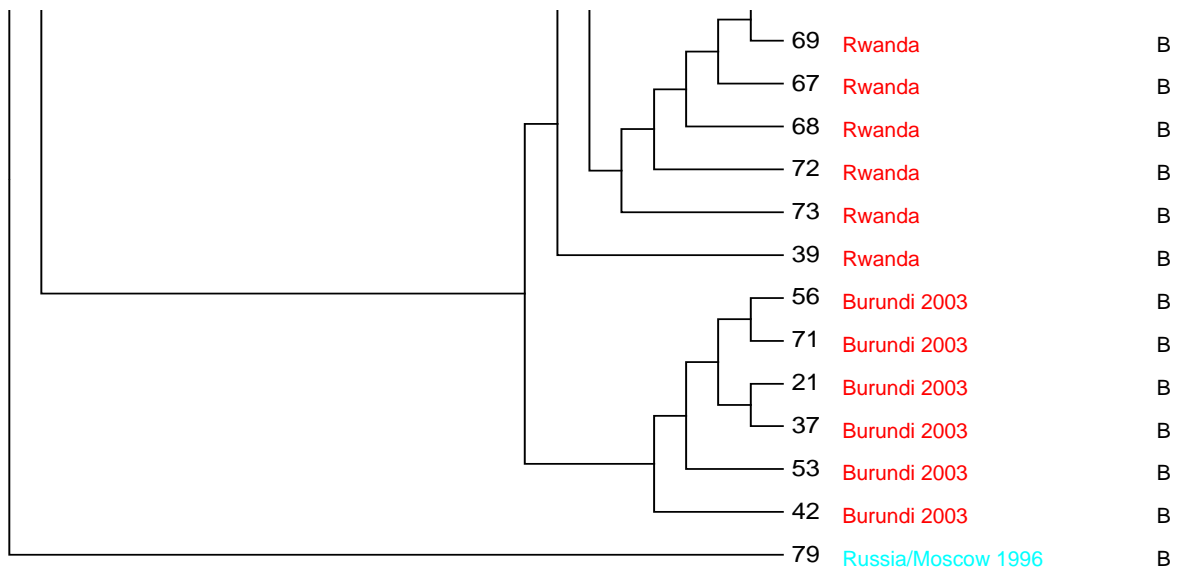

Supplement: Figure S5 — Phylogenetic organization of 97 human lice based on concatenation of four nuclear intergenic spacers, PM1, PM2, S2, and S5, using the Maximum parsimony method. (0.05 MB PDF) [file pntd.0000641.s005.pdf]
