# Supplementary material for: Genotyping of Human Lice Suggests Multiple Emergences of Body Lice from Local Head Louse Populations
Source: PLoS Negl Trop Dis. 2010 Mar 23;4(3):e641. doi: 10.1371/journal.pntd.0000641 (PMC2843630; doi:10.1371/journal.pntd.0000641)
Supplement: Figure S6 — Phylogenetic organization of 170 human lice based on partial sequence of the cytB gene, using the Maximum parsimony method. (0.02 MB PDF) [file pntd.0000641.s006.pdf]

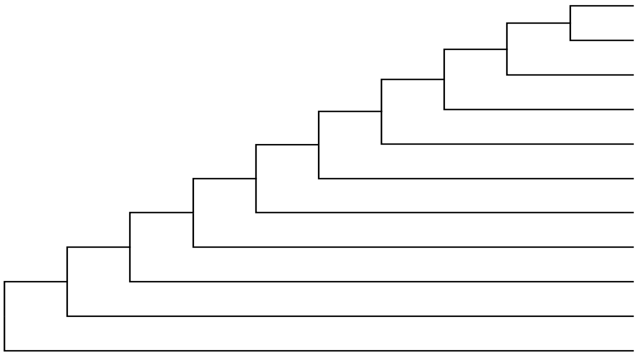

Sequence types	Origin year (number)	Body (B) or head (H)
4	*91 lice (see below)	H (6), B (85)
1	Moscow 1996 (1)	B
11	France	H
6	Russia 1997 (1), UK (4), USA (3)	H (7), B (1)
2	Mexico (8)	B
3	Moscow 1996 (1)	B
5	Moscow 1996 (1)	H
9	USA (2)	H
7	USA (12),	H
10	USA (19), UK (25)	H
8	Mexico (1)	H
